# Supplementary material for: The spillover effects of Medicare’s comprehensive care for joint replacement (CJR) model in California
Source: PLoS One. 2025 Apr 17;20(4):e0319582. doi: 10.1371/journal.pone.0319582 (PMC12005494; doi:10.1371/journal.pone.0319582)
Supplement: S1 File — (PDF) [file pone.0319582.s001.pdf]

S1 Table. Characteristics of All Patients Who Received Lower Extremity Joint Replacement in Treated and Control MSAs in California from 2014 to 2017 (N=411,004).

S1 Fig. Average Logged Adjusted Length of Stay of Patients Who Underwent Hip or Knee Joint Replacement in California from 2014 to 2017 (N=312,914).

S2 Fig. Average Proportion of Home Discharge of Patients Who Underwent Hip or Knee Joint Replacement in California from 2014 to 2017 (N=312,914).

S3 Fig. Time-trend Adjusted Changes in Logged Adjusted Length of Stay and Home Discharge Rates of Medicare Advantage Patients in Treated Relative to Control MSAs from 2014 to 2017 (N=83,277).

S2 Table. Time-trend Adjusted Changes in Logged Adjusted Length of Stay and Home Discharge Rates of Medicare Advantage Patients in Treated Relative to Control MSAs from 2014 to 2017 (N=83,277).

S3 Table. Comparison of the Difference-in-Differences Analyses Results of Traditional Medicare vs. Medicare Advantage vs. non-Medicare Patients without Age Restriction (N=410,978).

S4 Table. Time-trend Adjusted Changes in Logged Adjusted Length of Stay and Home Discharge Rates of Medicare Advantage Patients without Age Restriction in Treated Relative to Control MSAs from 2014 to 2017 (N=96,411).

S5 Table. Comparison of the Difference-in-Differences Analyses Results of Traditional Medicare vs. Medicare Advantage vs. non-Medicare Patients after Controlling for Admission Source (N=312,914).

S6 Table. Adjusted Changes in Logged Adjusted Length of Stay and Home Discharge Rates of Non-Medicare Patients in Treated Relative to Control MSAs from 2014 to 2017 after Controlling for Medicaid Beneficiaries (A) and Both Admission Source and Medicaid Beneficiaries (B) (N= 116,047).

S7 Table. Comparison of the Difference-in-Differences Analyses Results of Traditional Medicare vs. Medicare Advantage vs. non-Medicare Patients after Propensity Score Weighting (N=238,326).

S1 Table. Characteristics of All Patients Who Received Lower Extremity Joint Replacement in Treated and Control MSAs in California from 2014 to 2017 (N=411,004).

Characteristics	Total	Traditional Medicare	Medicare Advantage	Non-Medicare
No.	411,004	146,662	96,411	167,905
Age, mean (SD)	68.1 (11.1)	73.7 (8.7)	74.2 (8.1)	59.7 (8.9)
Women (%)	247,300 (60.2)	93,249 (63.6)	62,210 (64.5)	91,824 (54.7)
Race/ethnicity (%)				
White	290,812 (70.8)	111,874 (76.3)	67,789 (70.3)	111,136 (66.2)
Black	21,154 (5.2)	5,131 (3.5)	5,239 (5.4)	10,782 (6.4)
Hispanic	62,007 (15.1)	15,710 (10.7)	16,326 (16.9)	29,965 (17.9)
Asian / Pacific Islander	20,513 (5.0)	7,070 (4.8)	4,955 (5.1)	8,485 (5.1)
Native American / Eskimo / Aleut	1,072 (0.3)	338 (0.2)	205 (0.2)	529 (0.3)
Other or unknown	15,446 (3.8)	6,539 (4.5)	1,897 (2.0)	7,008 (4.2)
Diagnosis Related Group Code				
469, Major joint replacement with MCC (%)	15,943 (3.9)	8,454 (5.8)	4,341 (4.5)	3,147 (1.9)
470, Major joint replacement without MCC (%)	395,061 (96.1)	138,208 (94.2)	92,070 (95.5)	164,758 (98.1)

NOTE: MCC stands for Major Complication or Comorbidity. The total population includes patient records (n=26) that do not have information on primary payers (Traditional Medicare, Medicare Advantage, or non-Medicare).

S1 Fig. Average Logged Adjusted Length of Stay of Patients Who Underwent Hip or Knee Joint Replacement in California from 2014 to 2017 (N=312,914).

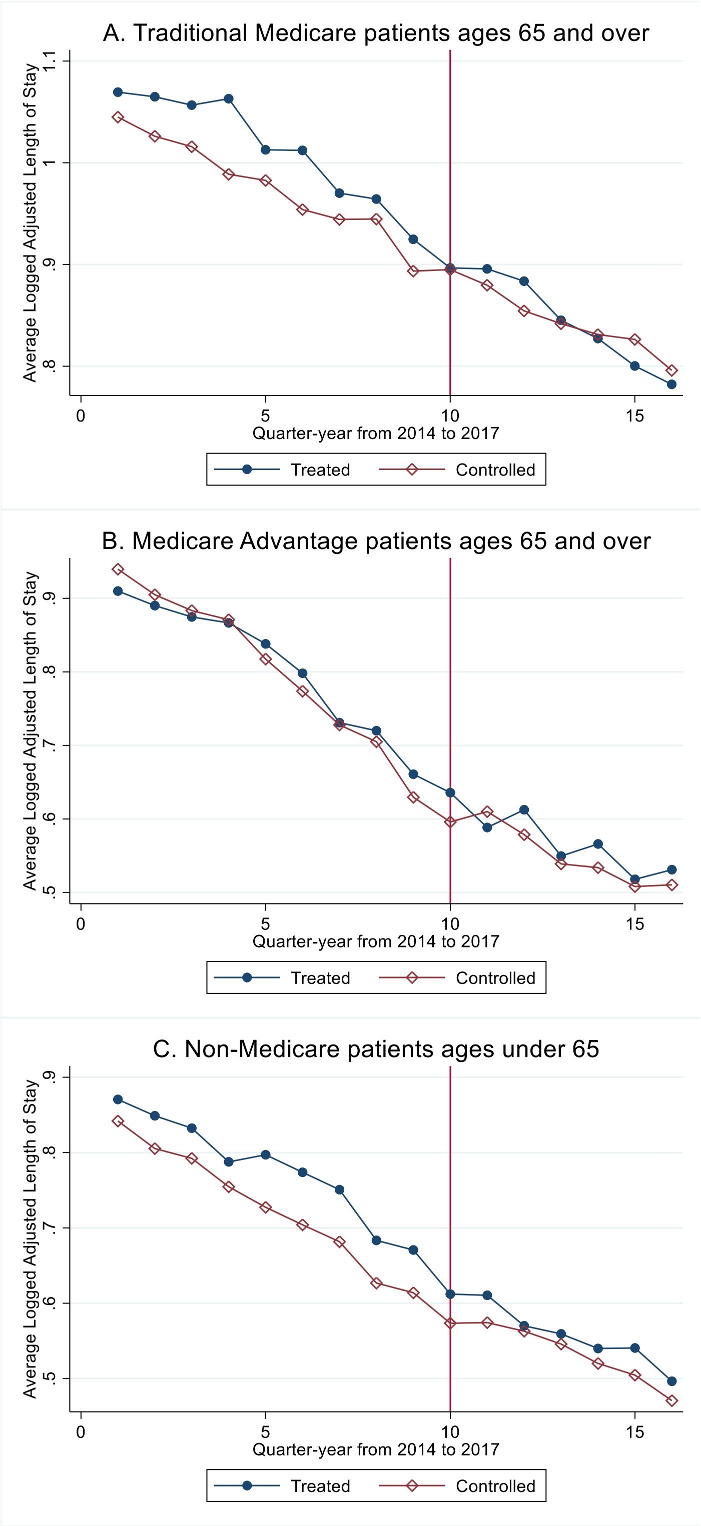

S2 Fig. Average Proportion of Home Discharge of Patients Who Underwent Hip or Knee Joint Replacement in California from 2014 to 2017 (N=312,914).

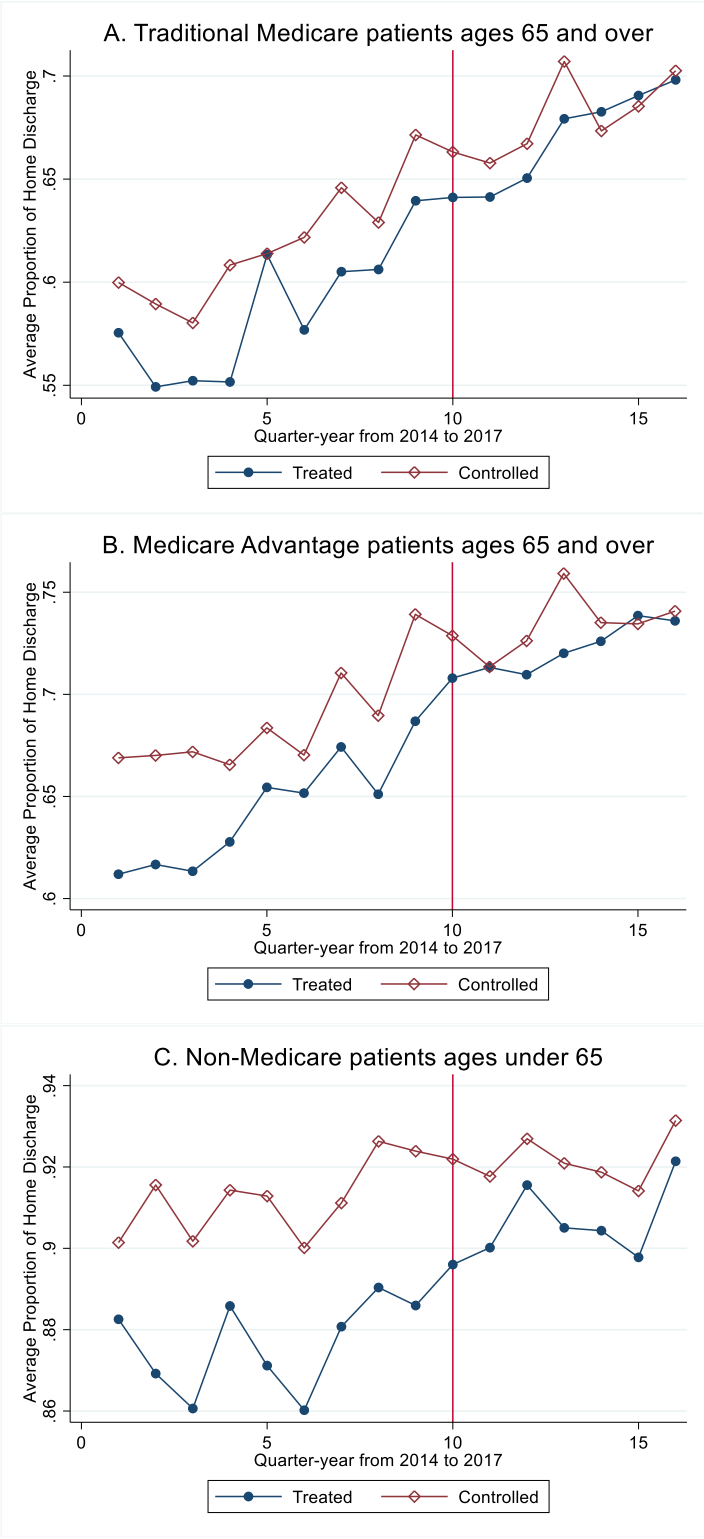

S3 Fig. Time-trend Adjusted Changes in Logged Adjusted Length of Stay and Home Discharge Rates of Medicare Advantage Patients in Treated Relative to Control MSAs from 2014 to 2017 (N=83,277).

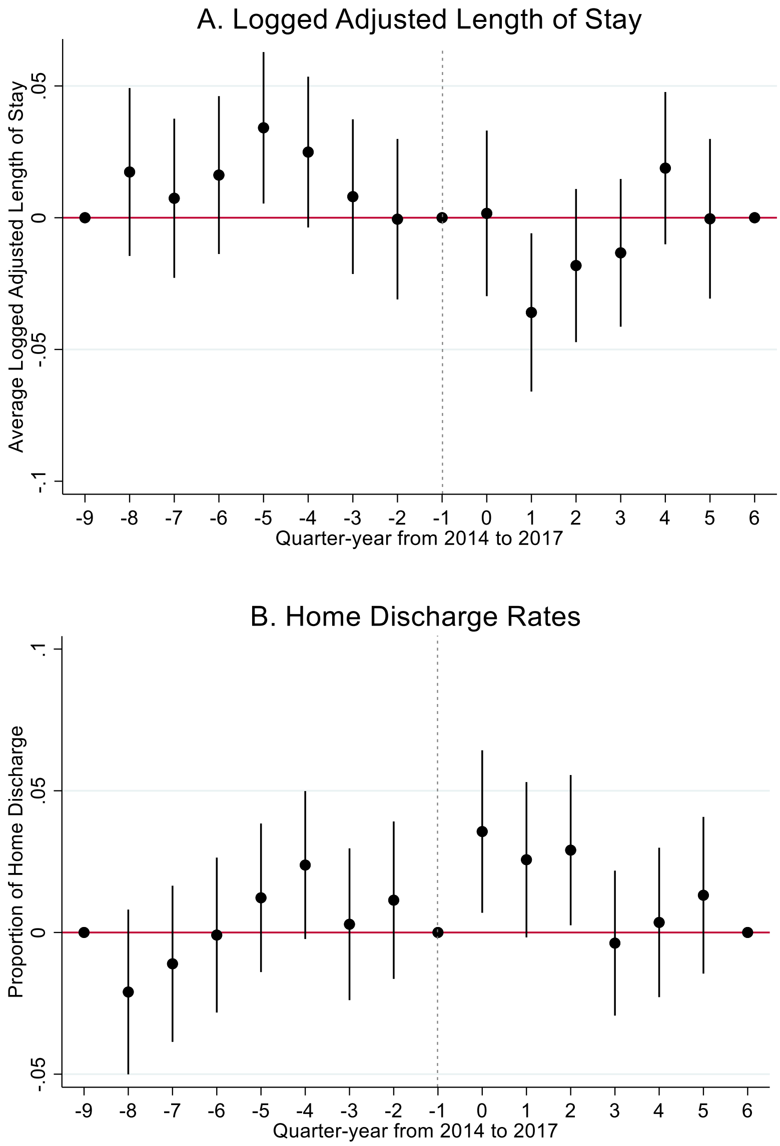

NOTE: 0 quarter-year represents the second quarter of 2016.

S2 Table. Time-trend Adjusted Changes in Logged Adjusted Length of Stay and Home Discharge Rates of Medicare Advantage Patients in Treated Relative to Control MSAs from 2014 to 2017 (N=83,277).

	Treated MSA		Control MSA		Difference in Differences			
	Pre-CJR	Post-CJR	Pre-CJR	Post-CJR	Coefficient (95% CI)	*P*	Relative Change, %	Wild Cluster Bootstrap
Logged Adjusted Length of Stay	0.81	0.57	0.80	0.55	-0.03 (-0.05, -0.00)	0.030	-3.0%	−0.08, 0.04
Home Discharge Rates	0.64	0.72	0.69	0.73	0.02 (-0.00, 0.05)	0.053	3.1%	−0.03, 0.05

S3 Table. Comparison of the Difference-in-Differences Analyses Results of Traditional Medicare vs. Medicare Advantage vs. non-Medicare Patients without Age Restriction (N=410,978).

		Treated MSA		Control MSA		Difference in Differences			
	**No. of Hospitalizations**	**Pre-CJR**	**Post-CJR**	**Pre-CJR**	**Post-CJR**	**Coefficient (95% CI)**	***P***	**Relative Change, %**	**Wild Cluster Bootstrap**
**Logged Adjusted Length of Stay**									
Traditional Medicare	146,662	1.02	0.86	0.98	0.85	-0.03 (-0.03, -0.02)	0.000	-3.0	−0.07, 0.00
Medicare Advantage	96,411	0.82	0.59	0.82	0.58	0.01 (0.00, 0.02)	0.015	1.0	−0.14, 0.15
Non-Medicare	167,905	0.82	0.61	0.76	0.58	-0.01 (-0.02, -0.00)	0.009	-1.0	−0.06, 0.02
**Home Discharge Rates**									
Traditional Medicare	146,662	0.58	0.67	0.61	0.68	0.03 (0.02, 0.04)	0.000	5.2	0.00, 0.06
Medicare Advantage	96,411	0.64	0.71	0.68	0.73	0.03 (0.02, 0.04)	0.000	4.7	−0.00, 0.07
Non-Medicare	167,905	0.83	0.88	0.88	0.89	0.02 (0.01, 0.02)	0.000	2.4	0.00, 0.04

S4 Table. Time-trend Adjusted Changes in Logged Adjusted Length of Stay and Home Discharge Rates of Medicare Advantage Patients without Age Restriction in Treated Relative to Control MSAs from 2014 to 2017 (N=96,411).

	**Treated MSA**		**Control MSA**		**Difference in Differences**			
	**Pre-CJR**	**Post-CJR**	**Pre-CJR**	**Post-CJR**	**Coefficient (95% CI)**	***P***	**Relative Change, %**	**Wild Cluster Bootstrap**
Logged Adjusted Length of Stay	0.82	0.59	0.82	0.58	-0.03 (-0.05, -0.01)	0.014	-3.0%	−0.08, 0.04
Home Discharge Rates	0.64	0.71	0.68	0.73	0.02 (-0.01, 0.04)	0.131	3.1%	−0.03, 0.04

S5 Table. Comparison of the Difference-in-Differences Analyses Results of Traditional Medicare vs. Medicare Advantage vs. non-Medicare Patients after Controlling for Admission Source (N=312,914).

		Treated MSA		Control MSA		Difference in Differences			
	**No. of Hospitalizations**	**Pre-CJR**	**Post-CJR**	**Pre-CJR**	**Post-CJR**	**Coefficient (95% CI)**	***P***	**Relative Change, %**	**Wild Cluster Bootstrap**
**Logged Adjusted Length of Stay**									
Traditional Medicare	113,590	1.02	0.86	0.98	0.85	-0.03 (-0.05, -0.03)	0.000	-3.0	−0.09, 0.00
Medicare Advantage	83,277	0.82	0.59	0.82	0.58	0.02 (0.01, 0.03)	0.002	2.0	−0.15, 0.17
Non-Medicare	116,047	0.82	0.61	0.76	0.58	-0.01 (-0.02, -0.00)	0.087	-1.0	−0.07, 0.03
**Home Discharge Rates**									
Traditional Medicare	113,590	0.58	0.67	0.61	0.68	0.02 (0.01, 0.03)	0.000	3.4	-0.00, 0.07
Medicare Advantage	83,277	0.64	0.71	0.68	0.73	0.03 (0.02, 0.04)	0.000	4.7	0.00, 0.08
Non-Medicare	116,047	0.83	0.88	0.88	0.89	0.01 (0.01, 0.02)	0.000	1.2	-0.00, 0.03

S6 Table. Adjusted Changes in Logged Adjusted Length of Stay and Home Discharge Rates of Non-Medicare Patients in Treated Relative to Control MSAs from 2014 to 2017 after Controlling for Medicaid Beneficiaries (A) and Both Admission Source and Medicaid Beneficiaries (B) (N= 116,047).

A. Controlling for Medicaid beneficiaries only

	**Treated MSA**		**Control MSA**		**Difference in Differences**			
	**Pre-CJR**	**Post-CJR**	**Pre-CJR**	**Post-CJR**	**Coefficient (95% CI)**	***P***	**Relative Change, %**	**Wild Cluster Bootstrap**
Logged Adjusted Length of Stay	0.78	0.56	0.72	0.54	-0.01 (-0.02, -0.00)	0.017	-1.0%	−0.08, 0.03
Home Discharge Rates	0.88	0.91	0.91	0.92	0.01 (0.01, 0.02)	0.000	1.1%	0.00, 0.03

B. Controlling for both admission source and Medicaid beneficiaries

	**Treated MSA**		**Control MSA**		**Difference in Differences**			
	**Pre-CJR**	**Post-CJR**	**Pre-CJR**	**Post-CJR**	**Coefficient (95% CI)**	***P***	**Relative Change, %**	**Wild Cluster Bootstrap**
Logged Adjusted Length of Stay	0.78	0.56	0.72	0.54	-0.01 (-0.02, 0.00)	0.118	-1.0%	−0.07, 0.04
Home Discharge Rates	0.88	0.91	0.91	0.92	0.01 (0.01, 0.02)	0.000	1.1%	-0.00, 0.03

S7 Table. Comparison of the Difference-in-Differences Analyses Results of Traditional Medicare vs. Medicare Advantage vs. non-Medicare Patients after Propensity Score Weighting (N=238,326).

		Treated MSA		Control MSA		Difference in Differences			
	No. of Hospitalizations	Pre-CJR	Post-CJR	Pre-CJR	Post-CJR	Coefficient (95% CI)	*P*	Relative Change, %	Wild Cluster Bootstrap
**Logged Adjusted Length of Stay**									
Traditional Medicare	86,812	0.99	0.86	0.96	0.85	-0.03 (-0.05, -0.02)	0.000	-3.0	−0.07, -0.01
Medicare Advantage	63,830	0.75	0.57	0.73	0.55	-0.00 (-0.02, 0.01)	0.548	-0.0	−0.13, 0.11
Non-Medicare	87,684	0.73	0.56	0.67	0.54	-0.03 (-0.04, -0.02)	0.000	-3.0	−0.08, 0.00
**Home Discharge Rates**									
Traditional Medicare	86,812	0.60	0.67	0.63	0.68	0.02 (0.01, 0.03)	0.000	3.4	-0.00, 0.07
Medicare Advantage	63,830	0.66	0.72	0.70	0.73	0.02 (0.01, 0.04)	0.000	3.0	0.01, 0.05
Non-Medicare	87,684	0.88	0.91	0.92	0.92	0.02 (0.01, 0.02)	0.000	2.3	0.00, 0.03

NOTE: All observations were divided into four groups based on treatment status and time period: pre-policy period & treated, pre-policy period & control, post-policy period & treated, and post-policy period & control. A multinomial logistic regression was then conducted to estimate the propensity of being in each group based on covariates. Subsequently, weights were calculated to correct for potential biases in group selection using the propensity scores. The weight for each observation was computed as the ratio of the predicted probability of being in group 1 (pre-policy period & treated) to the predicted probability of being in the other groups.
