# Supplementary material for: The spillover effects of Medicare’s comprehensive care for joint replacement (CJR) model in California
Source: PLoS One. 2025 Apr 17;20(4):e0319582. doi: 10.1371/journal.pone.0319582 (PMC12005494; doi:10.1371/journal.pone.0319582)
Supplement: Attachment — Submitted filename: PONE-D-23-30969 Response to Review.docx [file pone.0319582.s003.docx]

**Response to Review**

Re: Ms. No.: PONE-D-23-30969

Dear Reviewers,

Thank you for the opportunity to revise our manuscript “The Spillover Effects of Medicare’s Comprehensive Care for Joint Replacement (CJR) Model in California” for re-submission to *PLOS ONE*.

We have provided a point-by-point response and summary of revisions to each reviewer comment and editorial instruction below and highlighted all changes in the manuscript. We appreciate the reviewers’ feedback and suggestions to strengthen our paper.

Best,

Narae Kim and Mireille Jacobson

**Reviewer #1:**

1 The authors should first define what is the spillover effect.

Response: Thank you for this feedback. We acknowledge that the audience might not be familiar with thus concept. We added the definition of the spillover effect with an example in the introduction section.

2 There are many mainstream articles discussing spillover effects, but the authors did not mention any of them. Those mentioned are not CJR related papers.

Response: Thank you for this feedback. When we added the definition of spillover effect, we also added some references for readers who would like to learn more about the concept.

3 I am wondering since markers of quality of care were not affected, then why do the program still have spillover effects? Of course, I do not have an absolutely right answer. However, the authors should justify this in the introduction section.

Response: Thank you for your comments. We recognized that the introduction lacked sufficient explanation regarding what constitutes the 'spillover' effect of the program, aside from changes in quality of care. Therefore, we have included the definition of the spillover effects utilized in this study in the introduction: the 'spillover' effects are indirect outcomes of the policy beyond its intended target. In this study, we measured changes in inpatient length of stay and home discharge rates among patients not covered by traditional Medicare to verify the existence of spillover effects from the payment model.

4 The authors should apply propensity score matching to ensure that the distribution of every variable in different groups is identical and then present the results in a table.

Response: Thank you for your suggestions. For our main analysis, we used difference-in-differences methods, which accounts for both observable and unobservable differences that are on similar time paths between treatment and control groups and estimates the relative changes in outcomes of the treated group compared to control group after the policy implementation. Based on your suggestion, however, we acknowledge the concern of composition of treatment and control group systematically changing across time, affecting their trend and biasing the results. Therefore, we conducted additional propensity score weighting as a sensitivity analysis and added the results to our supplemental file. Importantly, the results are very similar when we employ the propensity score method.

5 How can the authors make sure there is a 2-year parallel trend before intervention when conducting the DID method? The authors have used some methods to assure parallel trend, but I do not know on which empirical studies the authors base them?

Response: Thank you for your feedback. We conducted a visual inspection based on unadjusted trend graphs (Supplementary Figure 1 & Supplementary Figure 2) and adjusted pre- and post-policy trend in the event study and DID analyses for MA patients as sensitivity analyses. However, we acknowledge the additional possibility of violation of parallel trend assumption based on several event study analyses results specifically in the year of 2014. Therefore, we limited the study period to 2015 to 2017 and added the results in Table 2 (Table 2 Panel B).

6 It is better to choose readmission, mortality, or infection rates as outcome measures since those three outcomes approximately belong to final outcomes. Moreover, several studies have already addressed them.

Response: Thank you for your suggestion. Unfortunately, our patient discharge dataset, which has detailed information on each hospitalization related to LEJR, has no further follow-up information. Thus, we do not have information regarding readmission, mortality, or infection rates. We discuss this in the limitations section. In addition, we intended to examine how hospitals responded to the policy implementation and how non-traditional Medicare patients were also affected by their behavioral change - patient treatment and care coordination - by measuring current outcomes of interest – length of hospital stay and discharge disposition.

**Reviewer #2**

1. In the study population, authors said that they excluded Medicare beneficiaries who are under age 65 because they might have different medical conditions. Then, I think authors also should exclude beneficiaries eligible for Medicare via End-Stage Renal Disease (ESRD) and Social Security Disability Insurance (SSDI) since they probably have different medical conditions that could bias the estimates.

Response: Thank you for your feedback. We agree that such conditions could bias our estimation; however, unfortunately, our dataset does not have the information. Instead, we controlled for MSDRG code to adjust for comorbidities and admission from emergency department to control for unobserved health conditions.

2. In the outcomes, is there any reason why authors did not include Skilled Nursing Facility (SNF) in the outcome variables? I think it is also important to add SNF because many previous works on CJR showed that CJR is associated with SNF.

Response: Thank you for your comments. We agree that discharge to SNF is also an important outcome variable dealt in prior studies. However, we decided to examine an increase in home discharge instead of examining a decrease in discharge to SNF as we can compare discharge to home vs. “institutional care,” which includes SNFs.

3. In the main analysis, did you use clustered standard error? It seems that patients might be subdivided into MSA level.

Response: Thank you for your comment. We did not use clustered standard errors at the MSA-level for our main analysis since this method can introduce bias and yield standard errors that are too conservative when the number of treatment clusters is too small. See page 10 for a discussion of this point. Instead, we used wild cluster bootstrap methods to address the concern of observations being clustered at a MSA-level and added the results in the main table (Table 2).

4. In figure 1 and 2, instead of labelling -9 to 6, it might be better to provide exact year and quarter such as Q1, 2014 and so on.

Response: Thank you for your feedback. We changed the labels of figure 1 and figure 2 following your suggestion.

5. It might be better if authors provide some details (e.g., covariates, standard errors, and so on) in table 2. Readers might be distracted without this information. Also, in table 2, authors should label the second column (e.g., The number of patients or N).

Response: Thank you for your feedback. We added the suggested information to Table 2 and label the second column following your suggestion.
