# Supplementary material for: The spillover effects of Medicare’s comprehensive care for joint replacement (CJR) model in California
Source: PLoS One. 2025 Apr 17;20(4):e0319582. doi: 10.1371/journal.pone.0319582 (PMC12005494; doi:10.1371/journal.pone.0319582)
Supplement: Attachment — Submitted filename: Point by point response_11102024.docx [file pone.0319582.s004.docx]

Dear Editor,

We sincerely appreciate your comments. We have revised the manuscript and tried our best to fully address the comments of the reviewers.

Details of the revision are given in the point-by-point response below. In addition, all the changes are tracked in the manuscript.

Warm regards,

Dr. Narae Kim & Dr. Mireille Jacobson

**Point-by-point response to reviewers:**

Reviewer #1:

1 It is recommended that relevant tables be included to present the differences in variable distribution before and after matching for patients in the experimental (CJR model) and control groups, in addition to the matching process. For instance, variables such as age could be compared to ensure similarity between the experimental and control groups after matching.

Response: Thank you for your comment. Table 1 shows the distribution of relevant variables (e.g., age, racial composition). Please refer to Table 1 for comparisons between groups (treatment vs. control; traditional Medicare vs. Medicare Advantage vs. non-Medicare; before vs. after policy implementation).

It's important to note that even though the distributions of relevant variables differ between treatment and control groups, these differences are not critical to the DID model, which rests on the assumption of similar or “parallel” trends in the pre-treatment period.

2 Many payment-related literature mentions the spillover effect. It is advised that the authors explore this phenomenon in the introduction. The authors should delineate the academic contributions of their article compared to previous works. For instance:
Glickman, S.W., et al. (2007), in their study titled "Pay for performance, quality of care, and outcomes in acute myocardial infarction," addressed the impact of pay-for-performance mechanisms on healthcare process and outcome, including untargeted measures
Urrusuno, R.F., et al. (2014), in "Compliance with quality prescribing indicators linked to financial incentives: what about not incentivized indicators?: an observational study," focused on the compliance with quality indicators and their association with financial incentives. However, the current study aims to extend this understanding by examining the spillover effects of payment policies on broader healthcare outcomes, thereby contributing to a more comprehensive understanding of payment mechanisms in healthcare.

Response: Thank you for your comment. While numerous studies have indeed examined spillover effects in pay-for-performance policies, it's important to note that the focus of Glickman et al. (2007) was primarily on the direct impact of the policy. Their study compared treatment and control hospitals, rather than specifically measuring spillover effects, even though non-targeted outcomes were included in their analysis.

Similarly, Urrusuno et al. (2014) did not concentrate on spillover effects. Instead, their research aimed to demonstrate the impact of financial incentives on prescribing quality indicators. They compared improvements in compliance between incentivized and non-incentivized indicators, concluding that the policy's impact on incentivized indicators was not straightforward.

In contrast, our study aligns more closely with the literature on spillover effects in the Comprehensive Joint Replacement (CJR) program. We've included several CJR studies with conflicting conclusions as our references. Our paper adds value to this ongoing debate by supporting one of these conclusions based on data from California, thus contributing to a more nuanced understanding of spillover effects in healthcare policy, specifically, the CJR program.

Reviewer #2: The authors have sufficiently addressed previous comments. As with every study, there are weaknesses, but the authors have acknowledged and addressed those weaknesses. The methodology is sound, and the topic is interesting and valuable.

Response: We appreciate your comment.

Reviewer #3: After reviewing the methods and results of this paper, I think the study design is generally appropriate. The use of both DID and event analysis ensures this study presents both the average effect and the micro-, quarterly effect of CJR implementation.

I have some further suggestions for the authors to consider:

1. Results presented should be interpreted carefully.

For example,
“TM patients hospitalized in treated MSAs had 0.04 fewer days or 3.9% lower adjusted length of stay compared to those in control MSAs (p<0.001),..” In this statement, the authors inaccurately interpreted the coefficient on marginal change in logged length of stay as marginal change in days (coefficient is -0.04).

Another example,
“In Table 2A, TM patients in treated MSAs had 0.02 percentage points or 3.4% higher home discharge rates compared to control MSAs after policy implementation (p<0.001).” Is the effect really 0.02 percentage points? If the probability is 0.02, then it should be 2 percentage points, not 0.02 percentage points (note: 0.02 percentage points is really small).

Also, I would recommend the authors to provide more details about how the relative change % was computed and what the meaning of this measure was.

Response: Thank you for your comment. We corrected the interpretation of the results – logged length of stay and home discharge rates. Changes are tracked in the manuscript.

2. To address concerns of clustering, the authors computed Wild Cluster Bootstrap CI. However, some of the results are somehow different from the main analysis. I would recommend authors to speculate reasons for such discrepancy. Did this prove that clustering at MSA level bias the estimates upward?

Response: Thank you for your comment. The wild bootstrap does not affect the point estimate but rather the standard errors and thus significance. As we now note in the paper on page 16, this discrepancy suggests that the estimated standard errors from the main analyses were understated, which is a common problem when using clustered standard errors in a setting with few treated clusters.

3. I would recommend the authors check numbers in all the tables (main tables and supplemental) and confirm the accuracy of those estimates. I found some of the DID coefficient does not match with the difference between pre-CJR and post-CJR between groups. In a linear setting (where the marginal effect on length of stay stays in logged form) I would assume the coefficients are differences between “within” and “between” differences.

Response: Thank you for your comment. We checked all the tables and confirmed their accuracy.

4. In the Results & Discussion section, the authors call out to Figure S4, but Figure S4 is not available.

Response: Thank you for your comment. We have correct this and changed it to S3.

5. The add-on propensity score method is interesting. The effect on logged length of stay turns non-significant after weighting. I would suggest the authors to provide a brief description about the PS methods used and how the weighting reduced sample size to 238,326.

Response: Thank you for your comment. The propensity score method was suggested by the other reviewer and added as part of a sensitivity analysis. The weighting did not reduce the sample size; we only applied the method to the 2015-2017 sample (n=238,326). The PS method did not show different results from the original analyses using 2015-2017 data. See Panel B of Table2.

We clarified that the PS method did not change the conclusions from the original analyses in the manuscript (p17) and added the detailed explanation of the PS weighting method we used in the supplemental file (S7 Table).
