# Supplementary material for: The spillover effects of Medicare’s comprehensive care for joint replacement (CJR) model in California
Source: PLoS One. 2025 Apr 17;20(4):e0319582. doi: 10.1371/journal.pone.0319582 (PMC12005494; doi:10.1371/journal.pone.0319582)
Supplement: Attachment — Submitted filename: Point by point response_01242025.docx [file pone.0319582.s005.docx]

Dear Editor,

We sincerely appreciate your comments. We have revised the manuscript and tried our best to fully address the comments of the reviewers.

Details of the revision are given in the point-by-point response below. In addition, all the changes are tracked in the manuscript.

Warm regards,

Dr. Narae Kim & Dr. Mireille Jacobson

**Point-by-point response to reviewers:**

1. Please change the subject “Results & Discussion” to “Results” on page 9.

Response: We changed the subject accordingly.

2. Why was the total number of hospitalizations (411,004) not equal to the sum (410,978) of the number of hospitalizations from Traditional Medicare (146,662), Medicare Advantage (96,411), and Non-Medicare(167,905) (S1 Table)?

Response: Thank you for the comments. The total population includes patient records (n=26) that lack primary payer information. To address potential confusion, we added a note to the table explaining this issue.

3. Please delete “log 0.04 or”, “log 0.01 or”, “log 0.02 or”, and “log 0.03 fewer days or” on page 13.

Response: We deleted the words accordingly.

4. Please move the sentence “This discrepancy suggests that the estimated standard errors from the main analyses were understated, which is a common problem when using clustered standard errors in a setting with few treated clusters (24)” on page 16 to the method section on page 9.

Response: We moved the sentence accordingly.
